# Complex *N*-Glycans Influence the Spatial Arrangement of Voltage Gated Potassium Channels in Membranes of Neuronal-Derived Cells

**DOI:** 10.1371/journal.pone.0137138

**Published:** 2015-09-08

**Authors:** M. Kristen Hall, Douglas A. Weidner, Michael A. J. Edwards, Ruth A. Schwalbe

**Affiliations:** 1 Department of Biochemistry and Molecular Biology, Brody School of Medicine at East Carolina University, Greenville, North Carolina, 27834, United States of America; 2 Department of Microbiology and Immunology, Brody School of Medicine at East Carolina University, Greenville, North Carolina, 27834, United States of America; Universidade de São Paulo, BRAZIL

## Abstract

The intrinsic electrical properties of a neuron depend on expression of voltage gated potassium (Kv) channel isoforms, as well as their distribution and density in the plasma membrane. Recently, we showed that N-glycosylation site occupancy of Kv3.1b modulated its placement in the cell body and neurites of a neuronal-derived cell line, B35 neuroblastoma cells. To extrapolate this mechanism to other N-glycosylated Kv channels, we evaluated the impact of *N*-glycosylation occupancy of Kv3.1a and Kv1.1 channels. Western blots revealed that wild type Kv3.1a and Kv1.1 α-subunits had complex and oligomannose N-glycans, respectively, and that abolishment of the N-glycosylation site(s) generated Kv proteins without N-glycans. Total internal reflection fluorescence microscopy images revealed that N-glycans of Kv3.1a contributed to its placement in the cell membrane while N-glycans had no effect on the distribution of Kv1.1. Based on particle analysis of EGFP-Kv proteins in the adhered membrane, glycosylated forms of Kv3.1a, Kv1.1, and Kv3.1b had differences in the number, size or density of Kv protein clusters in the cell membrane of neurites and cell body of B35 cells. Differences were also observed between the unglycosylated forms of the Kv proteins. Cell dissociation assays revealed that cell-cell adhesion was increased by the presence of complex N-glycans of Kv3.1a, like Kv3.1b, whereas cell adhesion was similar in the oligomannose and unglycosylated Kv1.1 subunit containing B35 cells. Our findings provide direct evidence that N-glycans of Kv3.1 splice variants contribute to the placement of these glycoproteins in the plasma membrane of neuronal-derived cells while those of Kv1.1 were absent. Further when the cell membrane distribution of the Kv channel was modified by N-glycans then the cell-cell adhesion properties were altered. Our study demonstrates that N-glycosylation of Kv3.1a, like Kv3.1b, provides a mechanism for the distribution of these proteins to the cell body and outgrowths and thereby can generate different voltage-dependent conductances in these membranes.

## Introduction

*N*-Glycosylation to newly synthesized membrane proteins is the most abundant protein co-translational modification in the lumen of the endoplasmic reticulum [1]. *N*-Linked oligosaccharides serve a number of important physiological roles including protein folding, protein assembly, and intracellular protein sorting, while outside the cell they are engaged in cell recognition events [2]. For instance, the presence of *N*-glycans attached to apical membrane proteins is critical for their trafficking to and retention in the apical membranes [3]. *N*-glycans have physiological roles in neuronal cells, and have been shown to significantly modulate electrical excitability [4]. It is of considerable importance to understand how the number, position, and type of N-glycans impact the spatial arrangement of membrane proteins in plasma membranes, which would further reinforce the role of glycans in membrane architecture [5].

Voltage-gated potassium channels (Kv) are critical components in the generation and propagation of electrical excitability in the nervous system [6]. The intrinsic electrical properties of a neuron depend on both the expression and plasma membrane distribution of Kv channel isoforms. A less defined role of Kv channels is their non-conducting functions, such as cell-cell interactions, cell migration, and cell proliferation [7]. The Kv potassium channels are the most diverse of the voltage-gated ion channel alpha subunits, with greater than 40 human genes in twelve subfamilies numbered Kv1 to Kv12 [8]. Ten of the twelve subfamilies consist of multiple members, and some of the members contain splice variants. There are two different carboxyl-terminus Kv3.1 splice variants (Kv3.1a, and —b) with Kv3.1b as the more abundant variant [9]. Both Kv3.1 splice variants have two absolutely conserved N-glycosylation sites running from amino acid residues 220 to 222 (NKT) and from 229 to 231 (NGT) [9]. Kv3.1b subunits are highly expressed in the mammalian brain in fast-spiking neurons such as neocortical and hippocampal interneurons as well as midbrain auditory neurons, while the shorter less abundant splice variant Kv3.1a is found in nonfast-spiking, somatostatin- and calbindin-containing interneurons [10]. The Kv1 channel family (except Kv1.6) has a single highly conserved N-glycosylation site between the first and second transmembrane segments [11]. The site runs from 207 to 209 (NTT) for Kv1.1. Kv1 channels are found predominantly on axons in the axonal membrane immediately preceding or within the axon nerve terminal, termed the axon initial segment [8].

Our previous studies showed that when either one or both of the *N*-glycosylation sites of the Kv3.1b channel were lost, the heterologously expressed ionic currents exhibited decreased activation, inactivation and deactivation rates, as well as reduced cell migratory rates [12]. Other studies have shown that Kv channels which have vacant N-glycosylation sites or lack sialic acid residues have altered channel gating [4,13]. We more recently demonstrated that N-glycosylation site occupancy of the Kv3.1b isoform modulated its placement in the cell body and neurites of a neuronal-derived cell line, B35 neuroblastoma cells [14]. Cellular assays demonstrated that the distinct spatial arrangements altered cell adhesion properties. We have here extended our studies on effects of N-glycosylation site occupancy to two other Kv channel members, namely Kv3.1a and Kv1.1. Western blot analysis demonstrated that the Kv1.1 alpha-subunit contained predominantly oligomannose N-glycans, in contrast to the complex N-glycans of Kv3.1a, as well as Kv3.1b [12]. We further show that the association of the Kv3.1a channel alpha-subunit with complex type N-glycans significantly influenced its localization to the outgrowth versus cell body while association of the Kv1.1 channel alpha-subunit with oligomannose type N-glycans did not appear to alter its spatial arrangement in the cell membrane. Also, the spatial arrangement was influenced by the protein content since the distribution of glycosylated forms of the Kv3.1a and Kv3.1b proteins were different and furthermore the unglycosylated forms of Kv3.1a, Kv3.1b, and Kv1.1 proteins had differences. This demonstrates that occupancy of the N-glycosylation site with complex N-glycans of the Kv3.1 splice variants provide a mechanism for the distribution of these proteins, thereby influencing voltage-dependent conductances in these membranes.

## Materials and Methods

### 2.1. Molecular Biology

Polymerase Chain Reaction (PCR) was employed to convert Asn residues at position 220 and 229 to Gln residues of Kv3.1a, as described for Kv3.1b [15]. PCR was also used to remove the stop codon and add BamHI sites at the 5’ and 3’ends of wild type and N220Q/N229Q Kv3.1a cDNAs, and also wild type and N207Q Kv1.1 cDNAs. All four cDNAs of correct DNA sequence were then cloned into BamHI (New England BioLabs, Ipswich, MA, USA) digested pEGFP-N3 (Clontech, Mountain View, CA, USA). Kv3.1a was a kind gift from Dr. Bernardo Rudy (New York University, New York, New York) [16]. Wild type and N207Q Kv1.1 cDNAs were a kind gift from Dr. William Thornhill (Fordham University, Bronx, NY) [17]. Standard procedures were followed for DNA sequencing, DNA amplification, DNA isolation, and subcloning [18].

### 2.2. B35 neuroblastoma cell culture and the establishment of stable cell lines

B35 neuroblastoma cells (rat central nervous system derived) were obtained from American Type Culture Collection (Manassas, VA, USA) and maintained in DMEM (Mediatech Inc., Manassas, VA, USA) supplemented with 10% fetal bovine serum (Invitrogen, Carlsbad, CA, USA), penicllin (50 U/mL) (Invitrogen, Carlsbad, CA, USA), and streptomycin (50 μg/mL) (Invitrogen, Carlsbad, CA, USA) at 37°C under 5% CO_2_ [19]. B35 cells were plated on uncoated 60 mm dishes (Fisher Scientific, Suwanee, GA, USA) every 3–4 days after a brief trypsin-EDTA (Invitrogen, Carlsbad, CA,USA) treatment and the cell culture medium was changed every 2–3 days. For the production of stable cell lines expressing the glycosylated and unglycosylated forms of the proteins, neomycin selectable pEGFN3 expression plasmids encoding wild type Kv3.1b, N220Q/N229Q Kv3.1b, Kv3.1a, N220Q/N229Q Kv3.1a, Kv1.1, and N207Q Kv1.1 proteins were transfected into B35 cells of 75%-80% confluency using Lipofectamine 2000 (Invitrogen, Carlsbad, CA, USA) according to the manufacturer’s protocol. In brief, FBS and antibiotic free DMEM (1mL) containing about 8 μg of recombinant vector and 15 μL of Lipofectamine 2000 (Invitrogen, Carlsbad, CA, USA) was added to each dish. Following an incubation of 5 h with the DMEM-DNA-lipid transfection solution, each dish of the B35 cells was re-fed with 3 mL of complete DMEM. Stable transfectants were selected by the addition of 1.0 g/L of Geneticin (Invitrogen, Carlsbad, CA, USA) to the cell culture medium. Cells expressing the various EGFP proteins were further enriched using a FACS Vantage (Becton Dickinson, Franklin Lakes, NJ, USA) cell sorter with laser excitation at 488 nm and green fluorescence emission at 515–545 nm. Isolated stable pools of transfected cells were then used for subsequent studies.

### 2.3. B35 total membrane isolation and glycosidase digestions

B35 cells expressing wild type, N220Q/N229Q Kv3.1a, Kv1.1, and N207Q Kv1.1 EGFP fusion proteins (about 1.35 X 10^8^) were homogenized (30–40 strokes) in 3 mL of lysis buffer (10 mM Tris (pH 7.4)); 250 mM sucrose, 5 mM EDTA; protease inhibitor cocktail set III 1:500 (Calbiochem, San Diego, USA). The homogenate was then centrifuged in an Eppendorf F-45-30-11 rotor (Eppendorf, Westbury, NY, USA) at 2,000 x g for 10 min at 4°C. Subsequently, the supernatant was transferred to an AH650 rotor (Sorvall, Newton, CT, USA) and centrifuged at 100,000 x g for 1 hr at 4°C. Lastly, the high speed pellet was resuspended in about 150 μl of lysis buffer and the protein concentration determined by Lowry method. Samples were stored at -80°C until needed.

Total B35 membranes (5g/L) containing wild type, N220Q/N229Q Kv3.1a, Kv1.1 or N207Q proteins fused to EGFP were treated with PNGase F (20 U/ μL) (New England Biolabs, Ipswich, MA, USA), Endo H (50 U/ μL) (New England Biolabs, Ipswich, MA, USA), or Neuraminidase (0.83 U/μL) (New England Biolabs, Ipswich, MA, USA) in NEB supplied buffers. Reactions were allowed to proceed overnight at 37°C and then stopped by addition of reducing SDS-PAGE sample buffer (2X).

### 2.4. Western blots

Reducing SDS sample buffer (2X) was added to B35 membranes and cell lysates for immunoblotting. Samples were electrophoresed at 20 mAmps for 105 min on 12% SDS gels. Electrophoresed proteins were transferred for 180 min at 250 mAmps onto Immobilon-P PVDF membranes (Millipore, Billercia, MA, USA). Blots were then incubated at room temperature for 20 min in blocking buffer on a rotating platform (PBS, 3% BSA with 0.1% Tween 20) followed by overnight incubation at 4°C in polyclonal rabbit anti-GFP antibody (Sigma, St. Louis, MO, USA) or monoclonal mouse anti-Kv1.1 (α-subunit) antibody (Neuromab, Davis, CA, USA). Next, blots were washed and incubated at room temperature for 1 hour with anti-rabbit or anti-mouse antibody conjugated to alkaline phosphate. ImmunO alkaline phosphatase substrate (MP Biomedicals, Irvine, CA, USA) was utilized to develop the immunobands.

### 2.5. TIRF microscopy

Stable transfected B35 cells expressing wild type Kv3.1b-EGFP, N220Q/N229Q Kv3.1b-EGFP, wild type Kv3.1a-EGFP, N220Q/N229Q Kv3.1a-EGFP, wild type Kv1.1-EGFP, N207Q Kv1.1-EGFP were seeded onto 35 mm poly-L-lysine coated glass bottom dishes (MatTek, Ashland, Ma, USA) and kept under culturing conditions for about 15–18h prior to imaging. Images were acquired as previously described, except the exposure time was 1500 ms [14,20,21]. In brief, images were excited with an argon laser beam of wavelength 488 nm entering the side illumination port of an Olympus 1X-71 microscope through a Apo 60X 1.45 objective and an ORCA R2 deep cooled mono CCD camera. Detection settings were kept constant for comparisons of the glycosylated and unglycosylated forms of Kv3.1a, Kv3.1b, and Kv1.1 proteins. The shutters, filters and camera, as well as data acquisition was controlled by Cell^TIRF Control 1.1 and Metamorph for Olympus Basic software. Image J software was used for mean data analysis of particles ranging from 5 to 10,000 pixels, as we previously described [14,20]. In brief, a particle represents the fluorescence intensity signal of EGFP tagged Kv protein. The number, size and mean fluorescence intensity signal of particles were determined for entire cell, cell body and each outgrowth. Origin 7.5 was used for graphics and statistics.

### 2.6. Dissociation assays

Equal densities of stable transfected B35 cells expressing wild type Kv3.1a-EGFP, N220Q/N229Q Kv3.1a-EGFP, wild type Kv1.1-EGFP, and N207Q Kv1.1-EGFP were seeded on 35 mm CellBind culture dishes (Corning, Corning, NY, USA), and then allowed to grow for 2 days. Confluent dishes were washed twice with PBS and fresh DMEM was added. A complete rotation with a cell scraper is used to detach cells and then cell aggregates were dissociated by pipetting up and down ten times. Images (30–35 fields/dish) were obtained on an Olympus IX 50 microscope using a 20X objective. Cell aggregates (≥ 2 cells/aggregate and >10 cells/ aggregate) were counted, and areas of cell clusters with more than 10 cells were determined using Image J software. The fraction of cell clusters is the number of clusters with more than 10 cells divided by the number of clusters with ≥2 cells for each image. The percent difference in cell clusters due to N-glycans was determined by taking the difference in fraction of cell clusters between glycosylated Kv (F_gly_) and unglycosylated Kv (F_ungly_), and dividing the difference by the fraction of cell clusters of unglycosylated Kv (F_ungly_) using the following equation: (*F*_*gly*_ ‒ *F*_*ungly*_)/*F*_ungly_*100. The percent difference in particle size due to N-glycans was determined in a similar manner, except the fraction (F) of cell cluster was substituted with particle size (S). Origin 7.5 was employed for graphics and statistics.

## Results and Discussion

### 3.1. Characterization of the Kv3.1a and Kv1.1 proteins with and without N-glycans

**Fig 1:**
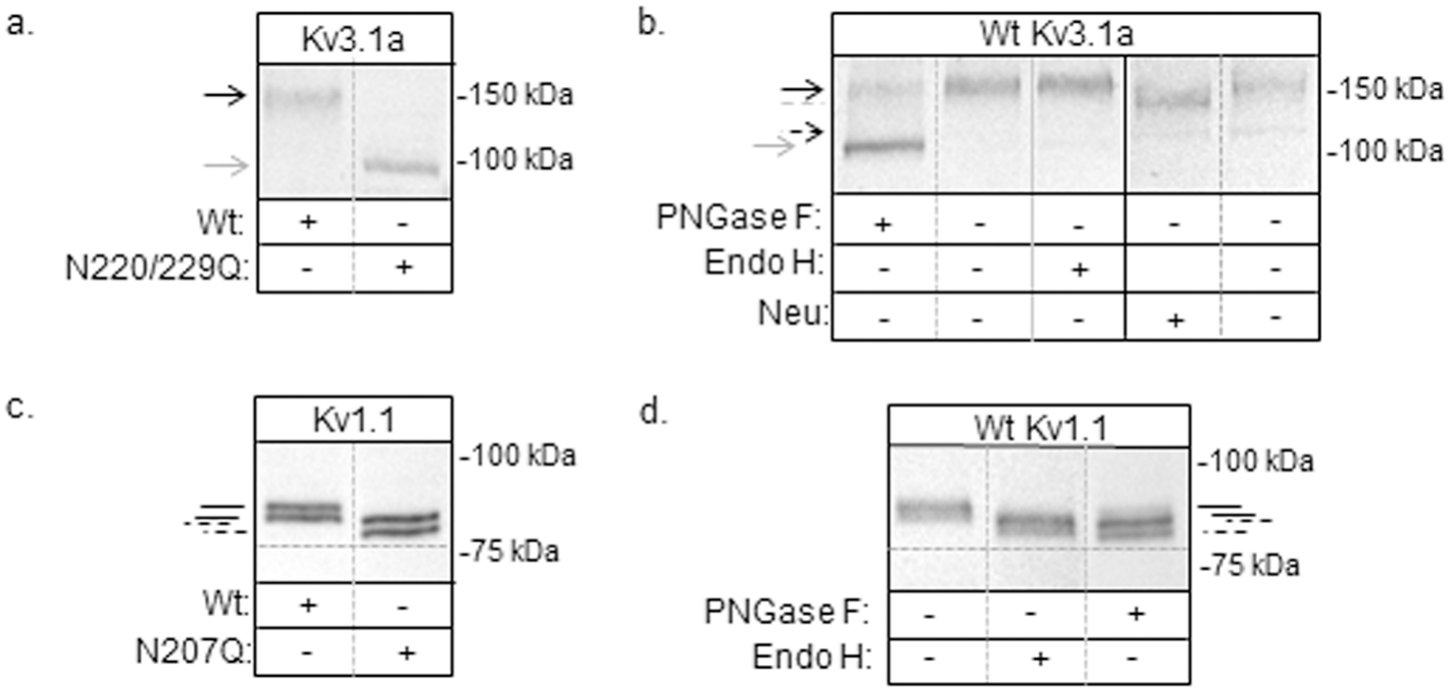
Characterization of glycosylated and unglycosylated Kv3.1a and Kv1.1 proteins heterologously expressed in B35 neuroblastoma cells. Western blots of wild type (Wt) and N220Q/N229Q Kv3.1a proteins from total membranes (a), and also Wt Kv3.1a protein samples digested (+) and undigested (-) with PNGase F, Endo H, or neuraminidase (neu), as indicated (b). Immunoblots of Wt and N207Q Kv1.1 proteins from transfected B35 cells (c), and also Wt Kv1.1 protein samples digested (+) and undigested (-) with glycosidases, as shown (d). Vertical solid grey line indicates a separate blot. The numbers adjacent to the Western blots represent the Kaleidoscope markers. Black arrows and black solid lines denote glycosylated wild type Kv proteins while grey arrows and black dashed lines represent unglycosylated Kv proteins. Grey dashed line signifies glycosylated Kv3.1a with sialic acid removed. Dashed arrow denotes Kv3.1a with oligomannose type N-glycans.

Previously, glycosylated (wild type), unglycosylated (N220Q/N229Q), and partially glycosylated (N220Q and N229Q) Kv3.1b α-subunit proteins were expressed in B35 neuroblastoma cells to show that *N*-glycan occupancy of the Kv3.1b glycoprotein could influence outward ionic current kinetics [12], sub-plasma membrane pools, and cellular properties [14]. Here we have utilized this same cultured cell model of central nervous system neurons [22] to ascertain whether N-glycosylation of other Kv channels (i.e. Kv3.1a and Kv1.1) provide a mechanism for modulating their spatial arrangement in the cell membrane and thereby cellular properties. The Kv3.1a protein is a Kv3.1 splice variant and like the Kv3.1b has two N-glycosylation sites in the first extracellular loop [9]. The Kv1.1 protein is a member of the Kv1 family and has a single N-glycosylation site in the first extracellular loop [23,24]. Further N-glycosylation of Kv1.1, like Kv3.1b [12] does not alter its stability and cell surface expression [25]. To begin, wt Kv3.1a and Kv1.1 proteins, along with their unglycosylated counterparts (i.e. N220Q/N229Q Kv3.1a and N207Q Kv1.1), were tagged with EGFP, and then N-glycosylation processing of the proteins were analyzed by Western blotting. Total membranes were obtained from B35 cells expressing wild type Kv3.1a, N220Q/N229Q Kv3.1a, wild type Kv1.1, and N207Q Kv1.1 proteins. A major immunoband was observed for wild type Kv3.1a (≈152 kDa), and N220Q/N229Q Kv3.1a (≈116 kDa) (Fig 1a). The increase in electrophoretic migration of the N220Q/N229Q Kv3.1a protein indicates that the N-glycosylation sites were disrupted. To characterize the type of N-glycans attached to Kv3.1a, glycosidase digestion reactions were utilized. Total membranes from B35 cells expressing wild type Kv3.1a were treated without (-) and with (+) PNGase F (removes complex, hybrid, and oligomannose *N*-glycans), Endo H (removes oligomannose and some hybrid *N*-glycans) or neuraminidase (cleaves sialyl residues from non-reducing termini of carbohydrate chains), and then analyzed by Western blotting (Fig 1b). The electrophoretic migrations were slowest for untreated and Endo H treated wild type Kv3.1a samples, and fastest for the sample treated with PNGase F. Further the sample treated with PNGase F migrated to a similar place as the N220Q/N229Q Kv3.1a protein. Treatment of Kv3.1a sample with neuraminidase migrated slightly faster than the untreated sample. As such, these results, along with our previous studies [26], indicate that the Kv3.1a protein has complex N-glycans at Asn220 and Asn229.

Doublets were detected for wild type (≈95 kDa and 92 kDa) and N207Q (≈91 kDa and 87 kDa) Kv1.1 proteins expressed in transfected B35 cells (Fig 1c). Further the type of N-glycan attached to Asn207 was oligomannose since treatment of total membranes with either PNGase F or Endo H shifted the immunobands to a similar position as those for the N207Q Kv1.1 protein (Fig 1d). The Kv1.1 protein doublet was also observed for wild type Kv1.1 heterologously expressed in mouse L-1 [23], Cos-1 [24], and CHO [25] cells, and in all cases it was reported that the type of N-glycan was oligomannose [23,24]. Further the studies suggested that the doublet was due to a different post-translational modification (e.g. phosphorylation, acetylation or O-glycosylation). However, these different modifications have yet to be clarified. It should also be mentioned that the Kv1.1 protein was reported to have complex N-glycans when expressed in certain isolated stable CHO cell lines [17]. Taken together, we will refer to wild type Kv3.1a and Kv1.1 proteins as glycosylated Kv proteins while the N220Q/N229Q Kv3.1a and N207Q Kv1.1 proteins will be termed unglycosylated Kv proteins. Additionally, heterologous expression of the Kv3.1a and Kv1.1 in B35 cells processes their N-glycans to complex and oligomannose types, respectively.

### 3.2. Role of N-glycans on subdomain localization of Kv proteins

**Fig 2:**
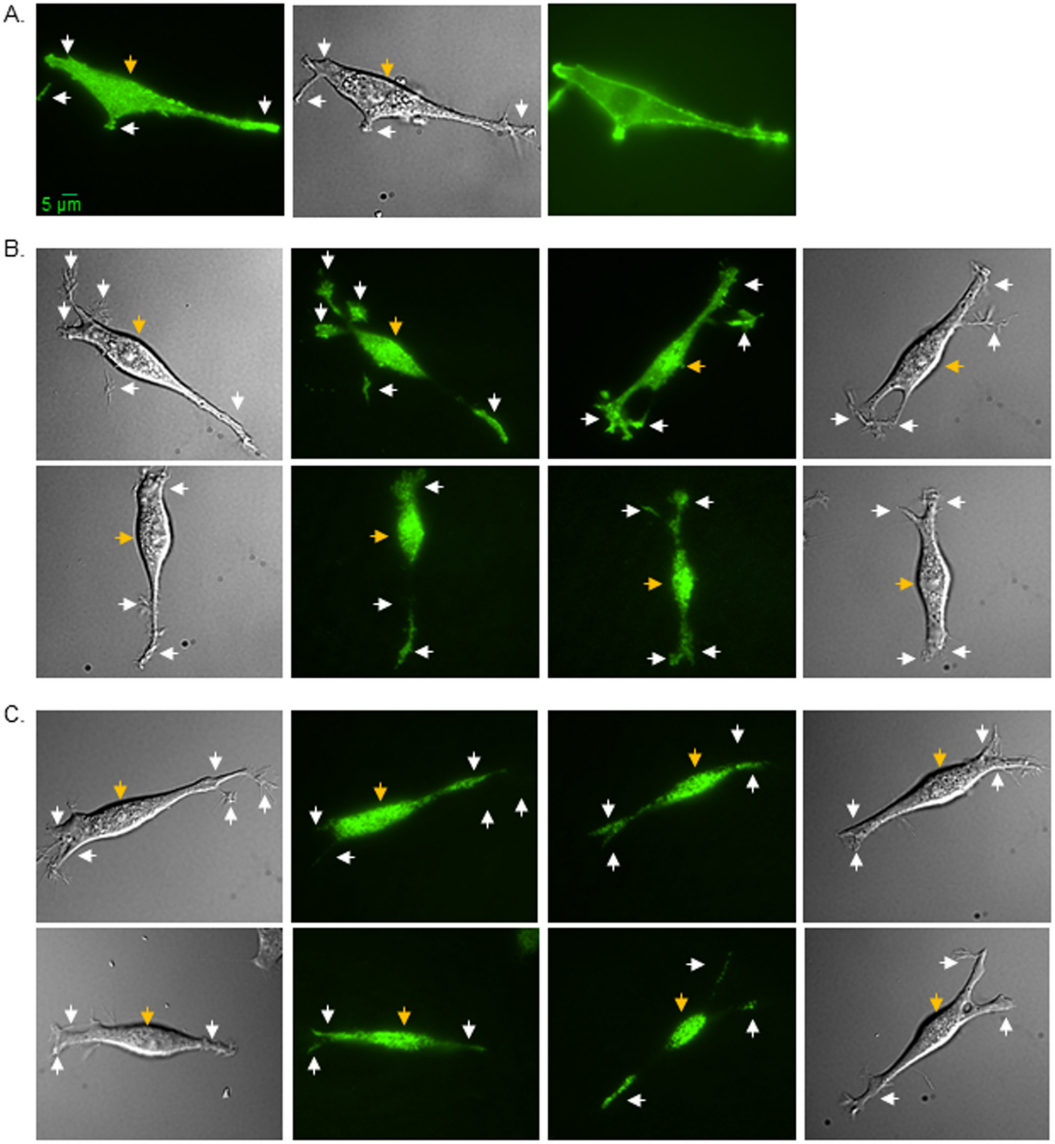
Spatial arrangement of glycosylated and unglycosylated forms of Kv proteins in B35 cells. Microscopy images were acquired in TIRF (left panel), DIC (middle panel), and wide-field (right panel) modes of EGFP tagged wild type Kv3.1b heterologously expressed in B35 cells (a). TIRF images (middle panels), along with their accompanied DIC images (outer panels), are shown for wild type Kv3.1a (b, upper row) and Kv1.1 (c, upper row) proteins, and also for N220Q/N229Q Kv3.1a (b, lower row) and N207Q Kv1.1 (c, lower row) proteins. Representative scale bar (5 μm) was identical for all images. White and gold arrows point to EGFP tagged Kv proteins in the outgrowth and cell body, respectively.

Recently, we showed that N-glycosylation of Kv3.1b provides a mechanism for its cell membrane distribution to the cell body and outgrowth of B35 neuroblastoma cells [14]. Since the protein expression level of Kv1.1 was significantly lower, the parameters to capture a TIRF image were different for this study. To establish that increasing the exposure time does not alter the quality of the TIRF images, TIRF (left panel), DIC (middle panel), and epifluorescence (right panel) images were acquired in the same plane for B35 cells transfected with glycosylated and unglycosylated Kv3.1b protein (Fig 2a). The signal to noise ratio of the protein in or near the adhered plasma membrane for the TIRF image is much higher than that of the epifluorescence image, indicating that images are being acquired in the TIRF mode. The DIC image accompanying the TIRF image is used as a reference to ensure the designation of the cell body and outgrowth for particle analysis. It should also be noted that to directly compare the distribution differences between glycosylated and unglycosylated Kv3.1b to those of glycosylated and unglycosylated forms of Kv3.1a and Kv1.1 proteins, microscopy images of Kv3.1b were acquired using virtually identical acquisition parameters. Representative TIRF and DIC images of glycosylated (upper panels) and unglycosylated (lower panels) Kv3.1a (Fig 2b) and Kv1.1 (Fig 2c) proteins are shown. The fluorescence intensity signal appeared much more intense in outgrowths for glycosylated Kv3.1a than its unglycosylated counterpart, as we previously observed for the Kv3.1b protein [14]. On the other hand, the fluorescence signal appeared much more concentrated in the cell body for B35 cells expressing Kv1.1 whether the glycosylation site was occupied or vacant. These results suggest that the N-glycans contribute to the spatial arrangement of the Kv3.1a protein in the cell membrane, similar to our finding with the Kv3.1b protein [14], while the N-glycan of Kv1.1 does not appear to influence its placement in the cell membrane.

**Fig 3:**
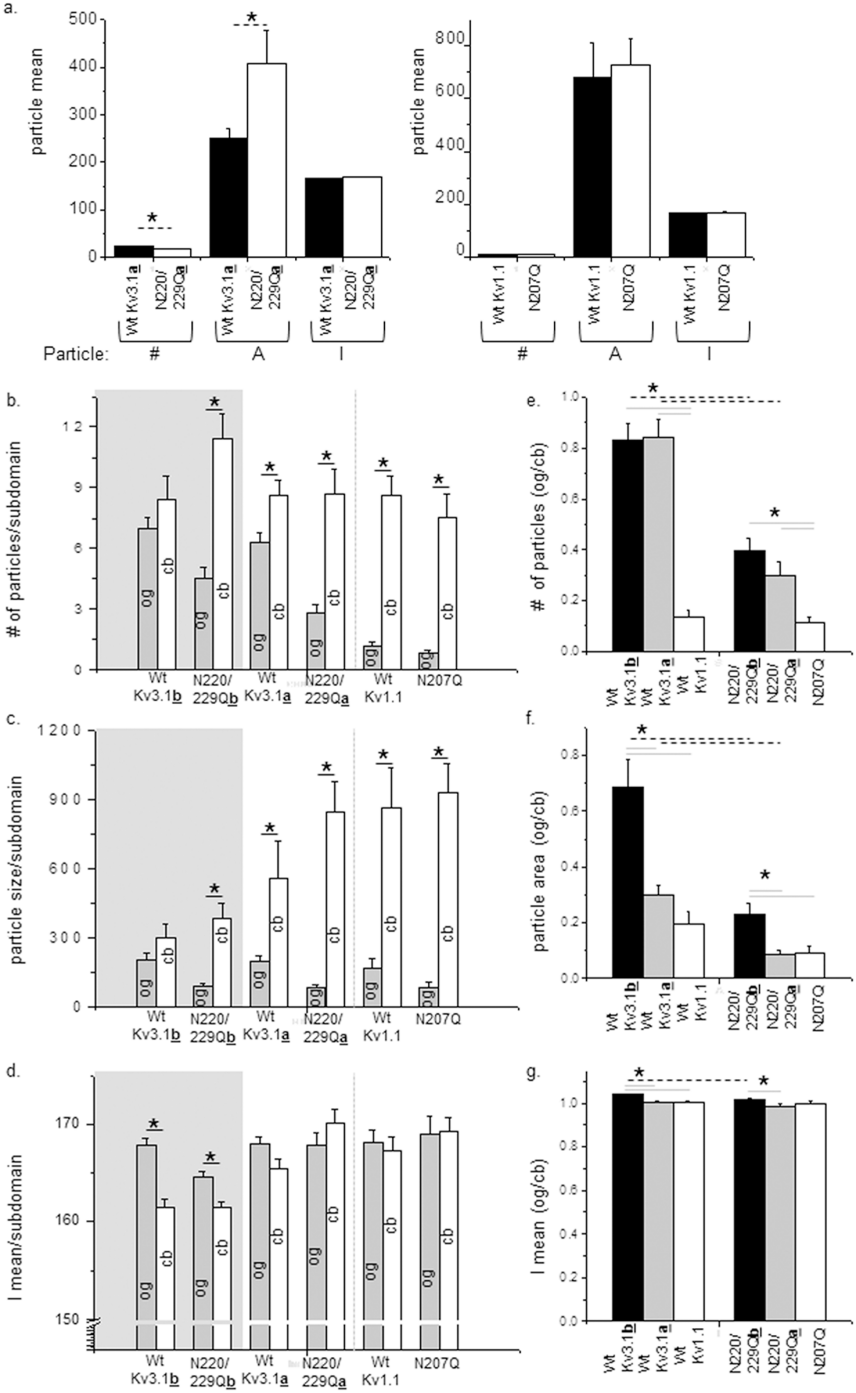
Particle analysis of glycosylated and unglycosylated forms of Kv channels in B35 cells. The particle number (#), area (A), and mean intensity (I) was determined for the total cell expressing glycosylated (wt Kv3.1a) and unglycosylated (N220/229Qa) Kv3.1a proteins or glycosylated (wt Kv1.1) and unglycosylated (N207Q) Kv1.1 proteins (a). The number of fluorescence particles (b), average size of those particles (c), and the mean intensity (d) was determined for cell body (cb) and outgrowth (og) for various forms of Kv3.1a, Kv3.1a and Kv1.1 proteins, as indicated. Experiments were conducted on 4 separate days for Kv3.1b and Kv3.1a, and 3 different days for Kv1.1. *n* denotes the number of cell bodies, and outgrowths (b, c and d) or the ratio of outgrowth (og) to cell body (cb) (e, f and g). Data are presented as the mean ± S.E. Mean differences were compared by student’s *t* test between glycosylated and unglycosylated forms of a given Kv channel or between different Kv proteins. Asterisks (*) indicate significant differences at a probability of 0.05. Dashed black lines represent differences between glycosylated Kv proteins and it unglycosylated counterpart. Solid black lines indicate differences between outgrowth and cell body of a Kv protein. Solid grey lines denote differences between either glycosylated Kv proteins or unglycosylated proteins.

Total fluorescence intensity signals of the various B35 cells reported that the amount of EGFP tagged Kv3.1a protein expressed by cells transfected with either glycosylated or unglycosylated Kv3.1a proteins were quite similar (Fig 3a, left panel). However, cells expressing the glycosylated Kv3.1a protein had a greater mean particle number and a smaller mean particle area than its unglycosylated counterpart. In terms of cells expressing the Kv1.1 protein, the glycosylated and unglycosylated forms were expressed at similar levels in the adhered membrane, and furthermore their mean particle values were virtually identical (Fig 3a, right panel). These results indicate that similar amounts of glycosylated and unglycosylated Kv proteins were expressed at the adherent plasma membrane, and therefore N-glycosylation processing is not required for protein folding. Further the results indicate that N-glycans modify the sub-plasma membrane pools of the Kv3.1a protein in B35 cells, as observed for Kv3.1b [14], while they do not appear to influence the distribution of the Kv1.1 protein.

Next, we evaluated whether the N-glycans of Kv3.1a or Kv1.1 proteins could alter the distribution of these Kv proteins to the cell body and outgrowth of B35 cells (Fig 3), as previously reported for Kv3.1b [14]. For a direct comparison of both Kv3.1a and Kv1.1 proteins to the Kv3.1b protein, data acquisition and analysis of glycosylated and unglycosylated forms of the Kv3.1b protein was conducted as well (Fig 3). Mean values for number (Fig 3b) and area (Fig 3c) of particles in the cell body for cells expressing glycosylated and unglycosylated forms of Kv3.1a were similar. However, mean values in the outgrowth of glycosylated Kv3.1a were roughly 2-fold greater than that of unglycosylated Kv3.1a. Cells expressing glycosylated or unglycosylated forms of the Kv1.1 protein had greater mean values for particle number and area in the cell body than outgrowth, and furthermore the values were quite similar between the two distinct forms (Fig 3c). The mean values of the mean intensity of the particle for the glycosylated and unglycosylated forms of either Kv3.1a or Kv1.1 proteins were quite similar in both domains (Fig 3d). The ratio of the mean values of outgrowth (og) to cell body (cb) for the glycosylated and unglycosylated forms of Kv3.1a and Kv1.1, as well as Kv3.1b, clearly illustrate the particle differences in the two forms for Kv3.1a and Kv3.1b, and the lack of change for the Kv1.1 (Fig 3e, 3f and 3g). These results, along with the Western blot results, indicate that occupancy of the sites of the Kv3.1a protein with complex type N-glycans provides a mechanism for modulating its distribution to the outgrowth and cell body while occupancy of the Kv1.1 protein with an oligomannose type N-glycan does not appear to influence its distribution in the cell membrane. Further the current study reveals that N-glycosylation processing of Kv3.1b alters its spatial arrangement in cell membranes with lower expression levels of Kv3.1b in B35 cells than the past study which analyzed cells with higher expression levels [14].

Bar graphs derived from the ratios of fluorescence intensity signals in outgrowth to that in cell body permitted us to directly compare the glycosylated Kv proteins, as well as the unglycosylated Kv proteins, to one another (Fig 3e, 3f and 3g). The particle number distribution between the subdomains for glycosylated Kv3.1a was quite similar to that of glycosylated Kv3.1b. However, there were considerable differences in the ratio of the size of the particles, as well as the mean intensity values of the particles between the glycosylated Kv3.1 splice variants. The differences in the ratios were largely due to quite similar particle area mean values for the subdomains of glycosylated Kv3.1b, and substantially larger particles in the cell body than outgrowth for glycosylated Kv3.1a. Further the density of the particles was greater in the outgrowth than cell body for glycosylated Kv3.1b while the density of the particle was not significantly different in the two subdomains for glycosylated Kv3.1a. These differences described for glycosylated Kv3.1a and Kv3.1b were also observed in their unglycosylated counterparts but to a lesser extent. In comparing glycosylated Kv1.1 to the glycosylated Kv3.1 splice variants, the ratio of the particle number in outgrowth versus cell body was much less for Kv1.1 than those for the Kv3.1 proteins. This was largely due to the very low number of particles in the outgrowths for Kv1.1. Ratios of the particle area and mean intensity of glycosylated Kv1.1 were quite similar to glycosylated Kv3.1a but smaller than those of glycosylated Kv3.1b. In terms of unglycosylated Kv proteins, ratios of the particle number for the unglycosylated Kv3.1 splice variants were different from that of unglycosylated Kv1.1. On the contrary, ratios of particle area and mean intensity were similar for unglycosylated Kv3.1a and Kv1.1 proteins but significantly different than those of unglycosylated Kv3.1b. These results indicate that the N-glycans, as well as the different C-termini, of the Kv3.1 splice variants, lead to differences in their distributions between the outgrowth and cell body. Results also reveal that the unglycosylated forms of Kv3.1a and Kv1.1 have protein clusters in the cell membrane of similar size and density but the number of protein clusters in the outgrowth relative to the cell body is significantly greater for unglycosylated Kv3.1a. Further when N-glycosylation sites of Kv3.1a were occupied with complex N-glycans and Kv1.1 with oligomannose N-glycans, considerable differences were introduced between the Kv3.1a and Kv1.1 proteins.

The lack of modulation of the Kv1.1 protein may be due to the absence of processing of the N-glycan to a higher order structure than an oligomannose type N-glycan. Of note, Western blots (Fig 1) revealed that the N-glycosylation site of the Kv1.1 protein was occupied by an oligomannose type N-glycan while Kv3.1a and Kv3.1b [12,19], had their sites occupied with complex type N-glycans. As such, it may be that occupancy of either hybrid or complex N-glycans is required to modify the spatial arrangement of the Kv1.1 protein in the membrane, not occupancy of the site by an oligomannose type N-glycan. Further oligomannose type N-glycans may have a lessened effect on the spatial arrangement of the Kv3.1 splice variants than complex type N-glycans. For instance, when the Kv3.1b protein was associated with complex N-glycans, instead of oligomannose N-glycans, the changes in the spatial arrangement of the Kv3.1b protein in the plasma membrane of CHO cells were much more visible [20]. It is also likely that the effect of the Kv3.1 variants with oligomannose N-glycans will be unalike since the spatial arrangement of the Kv3.1 variants with complex N-glycans had considerable differences. Overall, our results suggest that the segregation and clustering of N-glycosylated Kv3.1a and Kv3.1b proteins in neuronal membranes are strongly influenced by attachment of complex N-glycans, as well as the number of complex type *N*-glycans to Kv3.1b [14], and therefore support future studies in linking N-glycosylation site occupancy and N-glycan type to the spatial arrangement of N-glycosylated Kv proteins in the plasma membrane.

### 3.3. Cell-cell adhesion of glycosylated and unglycosylated forms Kv proteins expressed by B35 cells

**Fig 4:**
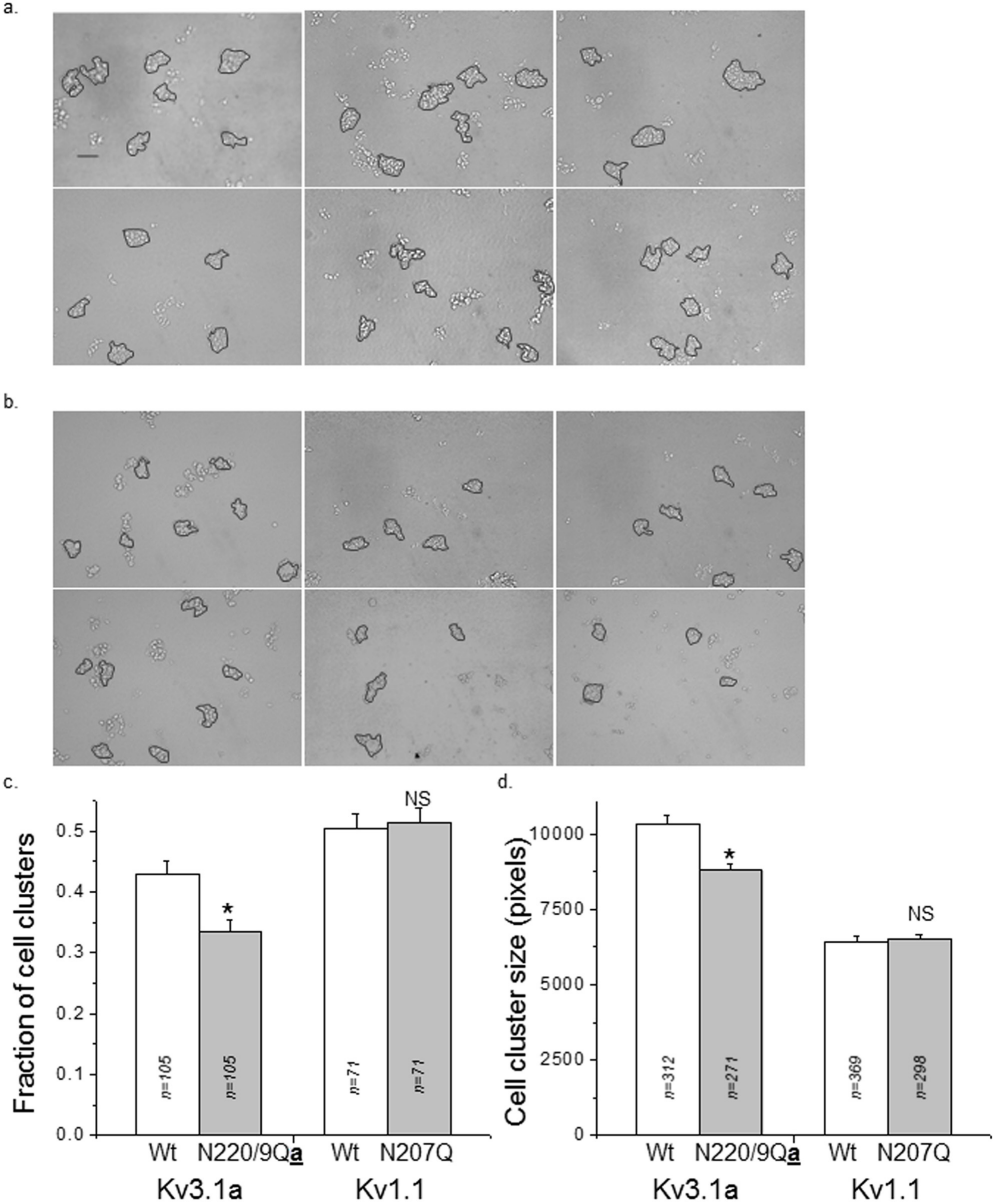
Cell dissociation assay of B35 cells transfected with glycosylated and unglycosylated forms Kv proteins. Representative microscopy images were acquired for wild type (upper panel) and N220Q/N229Q (lower panel) Kv3.1a (a) and also wild type (upper panel) and N207Q (lower panel) Kv1.1 transfected cells (b). The scale bar denotes 129 pixels or 0.25 μm. The percent of cell aggregates was calculated for particles comprised of >10 cells for glycosylated and unglycosylated forms of Kv3.1 and Kv1.1 proteins (c). Particle sizes were analyzed for cell aggregates with >10 cells (d). Data are presented as the mean ± S.E. and *n* denotes the number of images (c), and number of particles (d). Mean differences were compared by student’s *t* test. Asterisks indicate significant differences in mean values at a probability of 0.002, and NS signifies that the mean values were not significantly different at a probability of 0.05.

To determine whether changes in spatial arrangement of Kv proteins due to N-glycosylation occupancy correlated with modifications in cellular properties, we compared cell-cell interactions between glycosylated and unglycosylated forms of the Kv3.1a or Kv1.1 proteins. Representative images of cell clusters remaining after dissociation of a cell monolayer for transfected B35 cells with glycosylated (upper panel) and unglycosylated (lower panel) forms of the Kv3.1a (Fig 4a) and Kv1.1 (Fig 4b) proteins are shown. Cells expressing glycosylated Kv3.1a had a higher fraction of large cell clusters (>10 cells/cell cluster) than cells expressing unglycosylated Kv3.1a, while the fraction of large cell clusters generated by cells expressing glycosylated or unglycosylated forms of the Kv1.1 protein were unchanged (Fig 3c). Additionally, the size of the cell cluster (>10 cells/cell cluster) was significantly larger for cells expressing the glycosylated form of Kv3.1a than its unglycosylated counterpart, while the size of the cell clusters was similar for both forms of Kv1.1 (Fig 4d). These results indicate that changes in the spatial arrangement of the Kv protein in the plasma membrane due to N-glycosylation site occupancy correlate with changes in cell-cell adhesion.

Cell-cell adhesion properties of glycosylated and unglycosylated forms of the Kv3.1b protein were previously reported to be significantly different [14], similar to the result for the Kv3.1a protein. To compare the differences in the cell-cell adhesion properties of the Kv proteins, the percent difference in the cell-cell adhesion due to the N-glycans were determined for all three Kv proteins. The percent difference in cell clusters was about 42%, 29% and -1.6% for Kv3.1b, Kv3.1a and Kv1.1 proteins. The percent difference in cell cluster size was about 30%, 17% and -1.4% for Kv3.1b, Kv3.1a, and Kv1.1 proteins. These values suggest that the N-glycans of the Kv3.1b had a greater effect on the cell-cell adhesion properties than those of the Kv3.1a protein while the N-glycan of the Kv1.1 protein had no effect on cell-cell adhesion properties. The greater strengthening of adhesive contacts between neighboring cells for cells expressing Kv3.1b versus those expressing Kv3.1a correlate with greater levels and higher density of glycosylated Kv3.1b protein in the outgrowth than cell body. Further the absence of change in cell-cell adhesion as a function of oligomannose N-glycans for the Kv1.1 protein relates to the similar spatial arrangement patterns of the glycosylated and unglycosylated forms of the Kv1.1 protein in the cell membrane. Therefore, our findings support that the dissimilar spatial arrangements induced by the occupancy of the Kv3.1 splice variants with complex N-glycans are linked to changes in cellular properties of the B35 cells.

We have shown that N-glycans participate in membrane structure of neuronal membranes, since Kv3.1a and Kv3.1b protein clusters in the cell membrane differ as a result of occupancy of the N-glycosylation sites by complex type N-glycans. Further the differencesin spatial arrangement were related to differencesin cellular properties. We speculate that substitution of complex N-glycans with hybrid and oligomannose N-glycans will also influence the distribution of Kv3.1 splice variants to cell body and outgrowth since the activity of the glycosylated Kv3.1b channel with either oligomannose [15] or complex [12,13] type N-glycans was different from its unglycosylated counterpart. It is also likely that substitution of the N-glycan attached to Kv1.1 with a higher order N-glycan will induce changes in its spatial arrangement in the cell membrane as Kv1.1 channels associated with complex N-glycans had different channel gating than the Kv1.1 aglycoform [17]. This type of modulation of Kv channels by N-glycosylation is of considerable interest since altering the distribution of Kv channel to the cell body and neurites will have significant changes on voltage-dependent conductances of these membranes.
